# Laparoscopic Reduction and Repair of a Mesocolic Hernia Causing Small Bowel Obstruction: A Case Report and Review of Literature

**DOI:** 10.7759/cureus.37421

**Published:** 2023-04-11

**Authors:** Kamuran C Deger

**Affiliations:** 1 General Surgery, Biruni University Faculty of Medicine, Istanbul, TUR

**Keywords:** vomit, recurrent upper abdominal pain, gastrointestinal ileus, laparoscopic treatment, mesocolic hernia

## Abstract

Mesocolic hernias are a rare cause of small bowel obstruction that occurs when a loop of small bowel herniates through a defect in the mesocolon. We present a case of a 35-year-old male with a mesocolic hernia causing small bowel obstruction, who was successfully treated with laparoscopic reduction and repair. The patient had an uneventful recovery and was discharged on postoperative day 3. Mesocolic hernias should be considered in the differential diagnosis of small bowel obstruction, and prompt diagnosis and surgical intervention are essential to prevent complications such as bowel ischemia and perforation. Laparoscopic treatment can be a safe and effective option for the management of mesocolic hernias. This case report highlights the clinical presentation, radiological features, and surgical management of mesocolic hernias, with a focus on the role of laparoscopy in the treatment of this rare condition.

## Introduction

Internal hernias are a rare cause of small bowel obstruction. Mesocolic hernias are a subtype of internal hernias where the small bowel herniates through a defect in the mesocolon [1]. The right and left mesocolic hernias are the most encountered types of internal hernias. These hernias are different from ventral and inguinal hernias because the protrusion occurs inside the abdominal cavity rather than outside of it [2].

## Case presentation

Our emergency department received a 35-year-old male patient who complained of upper abdominal pain, nausea, and constipation. He mentioned that the pain had begun 14 days prior to his admission and had intensified over the past 48 hours. Furthermore, the patient had been experiencing mild cramping pain following meals for the past year. There was no history of any prior abdominal surgery. During the abdominal examination, the patient showed voluntary defense in the epigastric area. The laboratory test showed leukocytosis (white blood cell (WBC) of 11,200/mL) with mildly elevated C-reactive protein (CRP) level (10,20 mg/L). Upright abdominal X-ray showed air-fluid levels and distended small bowel loops. Next, the patient underwent a computed tomography (CT) scan of the abdomen with intravenous contrast (Video 1). This study revealed a 3 cm diameter defect in the mesentery, which may be compatible with a transmesenteric or intramesenteric small bowel internal hernia in the midline of the right half of the abdomen, and intestinal loops with a diameter of approximately 6 cm with a herniated area containing meso. Ileus was observed in the ileal and jejunal ans proximal to this localization. With these findings, the patient was taken to the operating room for laparoscopic exploration. The intestinal loops were entrapped through the right mesocolic defect. The small intestine was reduced from the hernia sac, and the defect was closed by interrupted silk sutures (Video 1). The patient had an uneventful postoperative recovery and was discharged from the hospital three days after the surgery. There were no complications, either in the early or late stages of the postoperative period.

**Video 1:** Right-sided mesocolic herniation treated totally with a laparoscopic approach

## Discussion

Mesocolic hernias are a rare subtype of internal hernias that occur when a loop of small bowel herniates through a defect in the mesocolon. The mesocolon is a double layer of peritoneum that attaches the colon to the posterior abdominal wall. Defects in the mesocolon can be congenital or acquired and may result from trauma, surgery, or inflammation [3]. There are three types of congenital mesocolic hernias. The first two are the right and left types, which make up 25% and 75% of all cases, respectively. The third type is extremely rare and known as the transverse mesocolic hernia.

Mesocolic hernias can present with symptoms of small bowel obstruction, such as abdominal pain, distention, and vomiting. Because the signs and symptoms are not specific, it needs a high index of clinical suspicion. Imaging studies, such as CT scans, can help identify the mesenteric defect and the herniated bowel loop [4].

Treatment is directed at the intestine to prevent obstruction and strangulation, which may lead to life-threatening complications, gangrene, and perforation. Surgical treatment is necessary to reduce the herniated bowel and repair the mesenteric defect. The defect can be closed primarily with interrupted or continuous sutures. We preferred to close the defects one by one with interrupted silk sutures, whereas Villalona et al. [5] preferred to close the defect in the sigmoid mesentery continuously with absorbable stitches [5]. Laparoscopic surgery has several benefits over traditional open surgery. It can result in less pain, shorter hospital stays, faster recovery, less scarring, reduced risk of infection, better visualization, and reduced bleeding. Over the past few years, there have been reports indicating that laparoscopic treatment can be a safe and feasible surgical alternative for managing mesocolic hernias in suitable patients [6,7]. Thus, we also preferred to perform the treatment of our patient via a laparoscopic approach.

## Conclusions

Mesocolic hernias are a rare cause of small bowel obstruction but should be considered in the differential diagnosis. Prompt diagnosis and surgical intervention are essential to prevent complications such as bowel ischemia and perforation. Laparoscopic treatment can be a safe and effective option for the management of mesocolic hernias.
